# Supplementary material for: The cauliflower mosaic virus transmission helper protein P2 modifies directly the probing behavior of the aphid vector Myzus persicae to facilitate transmission
Source: PLoS Pathog. 2023 Feb 6;19(2):e1011161. doi: 10.1371/journal.ppat.1011161 (PMC9934384; doi:10.1371/journal.ppat.1011161)
Supplement: S1 Fig — Kinetics of symptom onset on turnip plants after mechanical inoculation with leaf extracts revealed no significant differences between JI and JIΔP2, neither for the percentage of infected plants (N = 29–30) (Pearson’s Chi-squared test, χ2 = 0.01, Df = 1, p = 0.92) nor the day of symptom onset (Wilcoxon rank sum test, W = 437, p = 0.970). (PDF) [file ppat.1011161.s001.pdf]

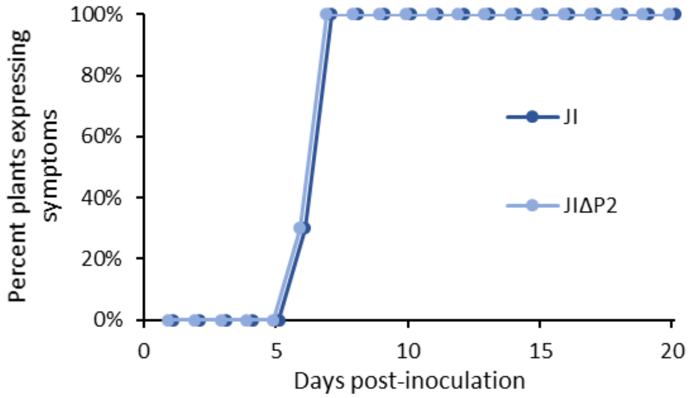

S1 Fig. Kinetics of symptom onset on turnip plants after mechanical inoculation with leaf extracts revealed no significant differences between JI and JIΔP2, neither for the percentage of infected plants (N = 29 - 30) (Pearson's Chi-squared test, $\chi^2 = 0.01$, Df = 1, p = 0.92) nor the day of symptom onset (Wilcoxon rank sum test, W = 437, p = 0.970).
