# Supplementary material for: The cauliflower mosaic virus transmission helper protein P2 modifies directly the probing behavior of the aphid vector Myzus persicae to facilitate transmission
Source: PLoS Pathog. 2023 Feb 6;19(2):e1011161. doi: 10.1371/journal.ppat.1011161 (PMC9934384; doi:10.1371/journal.ppat.1011161)
Supplement: S2 Fig — (a) Instant Blue stained protein gel after SDS-PAGE. The slots were loaded with the indicated components. (b) Western blot analysis of purified recombinant his-tagged P2 (HP2), partially purified recombinant P3, and purified virus particles. The blots were developed with the indicated antisera. (PDF) [file ppat.1011161.s002.pdf]

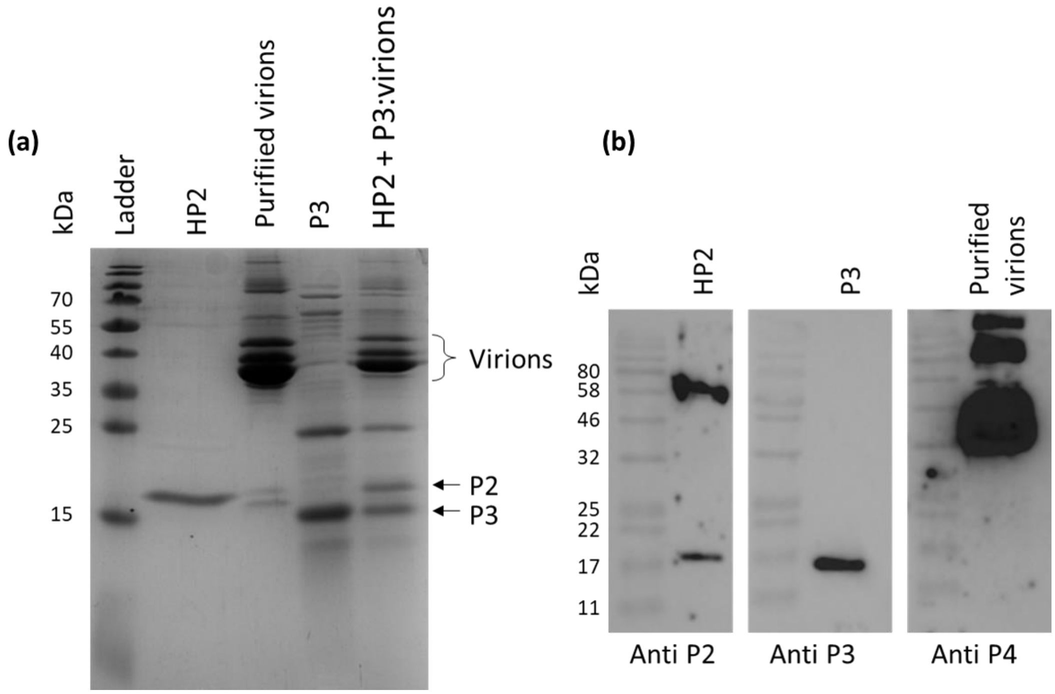

S2 Fig. Viral components used for aphid feeding assays on artificial medium. (a) Instant Blue stained protein gel after SDS-PAGE. The slots were loaded with the indicated components. (b) Western blot analysis of purified recombinant his-tagged P2 (HP2), partially purified recombinant P3, and purified virus particles. The blots were developed with the indicated antisera.
