# Supplementary material for: The cauliflower mosaic virus transmission helper protein P2 modifies directly the probing behavior of the aphid vector Myzus persicae to facilitate transmission
Source: PLoS Pathog. 2023 Feb 6;19(2):e1011161. doi: 10.1371/journal.ppat.1011161 (PMC9934384; doi:10.1371/journal.ppat.1011161)
Supplement: S3 Fig — Bars show means and standard errors. Before recording aphid feeding behavior on healthy plants, aphids were allowed to feed under EPG control for 1 h on different artificial media: 15% sucrose in DB5 buffer (light grey); DB5 buffer alone (orange) or DB5 buffer supplemented with P3 and purified virus particles (P3:virions, dark grey); his-tagged P2 (HP2, yellow); or HP2 and P3:virions (blue). Viral components used for aphid feeding assays on artificial medium are shown in S3 Fig. Only aphids having inserted their stylets for at least 5 min in the artificial media were used for the experiments (N = 21–26). EPG parameters related to duration are displayed. Letters indicate significant differences between artificial media as tested by GLM (generalized linear model) followed by pairwise comparisons using “emmeans” (p < 0.05 method: Tukey). (PDF) [file ppat.1011161.s003.pdf]

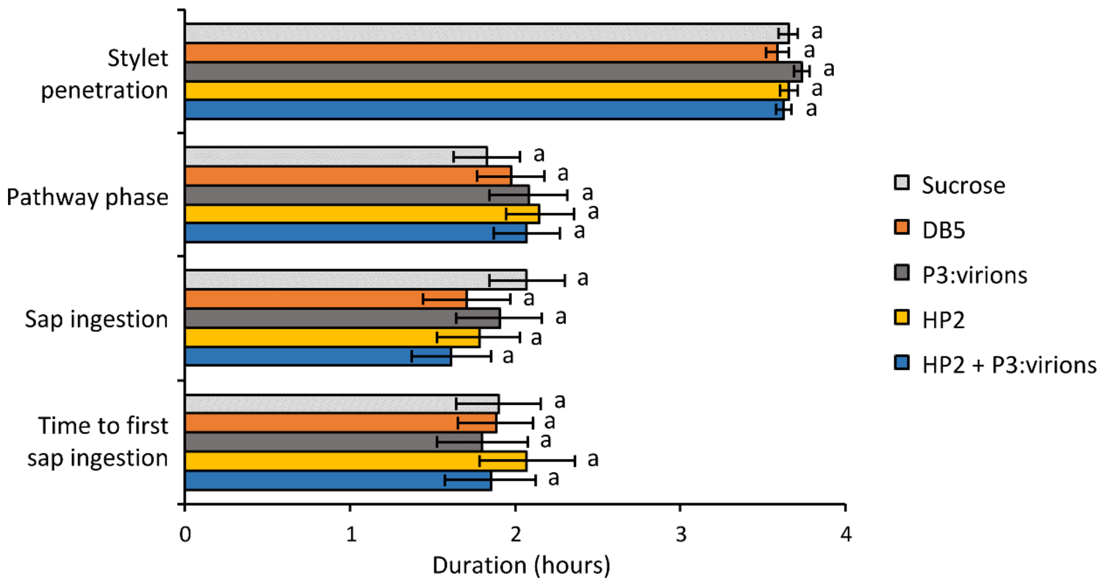

S3 Fig. Feeding behavior of *Myzus persicae* on healthy plants after membrane acquisition of P2 and P3:virions. Bars show means and standard errors. Before recording aphid feeding behavior on healthy plants, aphids were allowed to feed under EPG control for 1 h on different artificial media: 15 % sucrose in DB5 buffer (light grey); DB5 buffer alone (orange) or DB5 buffer supplemented with P3 and purified virus particles (P3:virions, dark grey); his-tagged P2 (HP2, yellow); or HP2 and P3:virions (blue). Viral components used for aphid feeding assays on artificial medium are shown in supplementary Figure 3. Only aphids having inserted their stylets for at least 5 min in the artificial media were used for the experiments (N = 21-26). EPG parameters related to duration are displayed. Letters indicate significant differences between artificial media as tested by GLM (generalized linear model) followed by pairwise comparisons using “emmeans” ($p < 0.05$ method: Tukey).
