# Supplementary material for: The cauliflower mosaic virus transmission helper protein P2 modifies directly the probing behavior of the aphid vector Myzus persicae to facilitate transmission
Source: PLoS Pathog. 2023 Feb 6;19(2):e1011161. doi: 10.1371/journal.ppat.1011161 (PMC9934384; doi:10.1371/journal.ppat.1011161)
Supplement: S4 Fig — Bars show means and standard errors. The behavior of individual aphids was recorded by electrical penetration graph (EPG) for 4 h (N = 26–28). EPG parameters related to duration are displayed. Different letters indicate significant differences between plant infection status as tested by GLM (generalized linear model) followed by pairwise comparisons using “emmeans” (p < 0.05 method: Tukey). (PDF) [file ppat.1011161.s004.pdf]

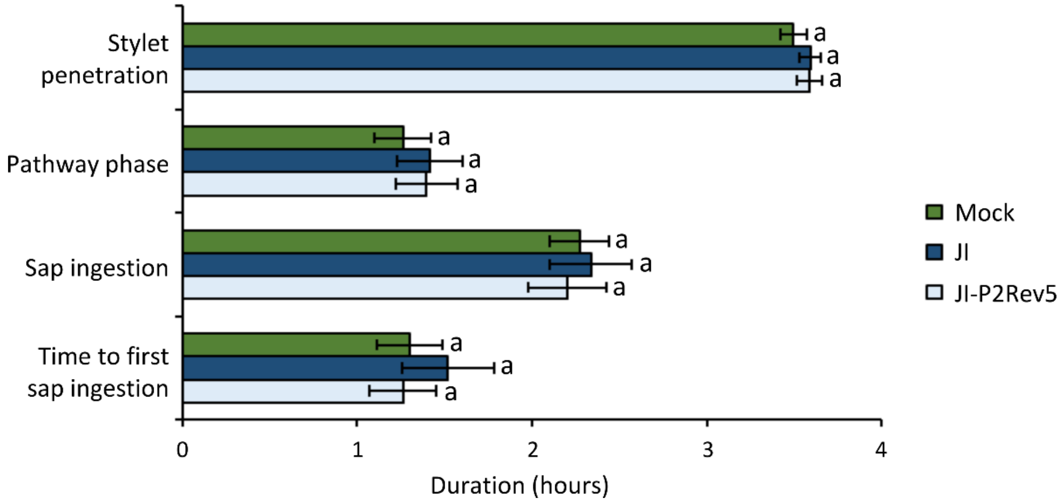

S4 Fig. Feeding behavior of *Myzus persicae* on mock-inoculated, JI- or JI-P2Rev5-infected turnip plants at 14 dpi. Bars show means and standard errors. The behavior of individual aphids was recorded by electrical penetration graph (EPG) for 4 h (N = 26-28). EPG parameters related to duration are displayed. Different letters indicate significant differences between plant infection status as tested by GLM (generalized linear model) followed by pairwise comparisons using “emmeans” ($p < 0.05$ method: Tukey).
