# Supplementary material for: The cauliflower mosaic virus transmission helper protein P2 modifies directly the probing behavior of the aphid vector Myzus persicae to facilitate transmission
Source: PLoS Pathog. 2023 Feb 6;19(2):e1011161. doi: 10.1371/journal.ppat.1011161 (PMC9934384; doi:10.1371/journal.ppat.1011161)
Supplement: S5 Fig — Bars show means and standard errors. (a-b) Feeding behavior of Myzus persicae on healthy plants after membrane acquisition of wild type P2 and P3:virions (P2+P3:virions), P2 carrying the mutation Rev5 and P3:virions (P2Rev5+P3:virions) or of an irrelevant protein (CLINK). Before recording aphid feeding behavior on healthy plants, aphids were allowed to feed under EPG control for 1 h on the different artificial media. Only aphids having inserted their stylets for at least 5 min in the artificial media were used for the experiments (N = 29–34). Selected EPG parameters are presented sorted according to (a) duration or (b) occurrence. Different letters show significant differences between treatments as tested by GLM (generalized linear model) followed by pairwise comparisons using “emmeans” (p < 0.05 method: Tukey). (a) Statistical analysis of the duration of events revealed no difference for parameters shown (GLM, p > 0.05). (b) Statistical analysis of the occurrence of events revealed significant differences for the numbers of intracellular punctures during first penetration and total intracellular punctures (GLM, Df = 2, χ2 = 95.247, χ2 = 49.683, p < 0.001, respectively). None of the other statistically processed parameters showed a significant difference between treatment (see S5 Tables). (c) Instant Blue stained protein gel after SDS-PAGE of Sf9 crude extracts used for aphid feeding assays on artificial medium. Black arrows point to the position of P2 and P2Rev5, P2:GFP and P2Rev5:GFP, and CLINK, respectively. P2:GFP and P2Rev5:GFP were not used in our experiment. (PDF) [file ppat.1011161.s005.pdf]

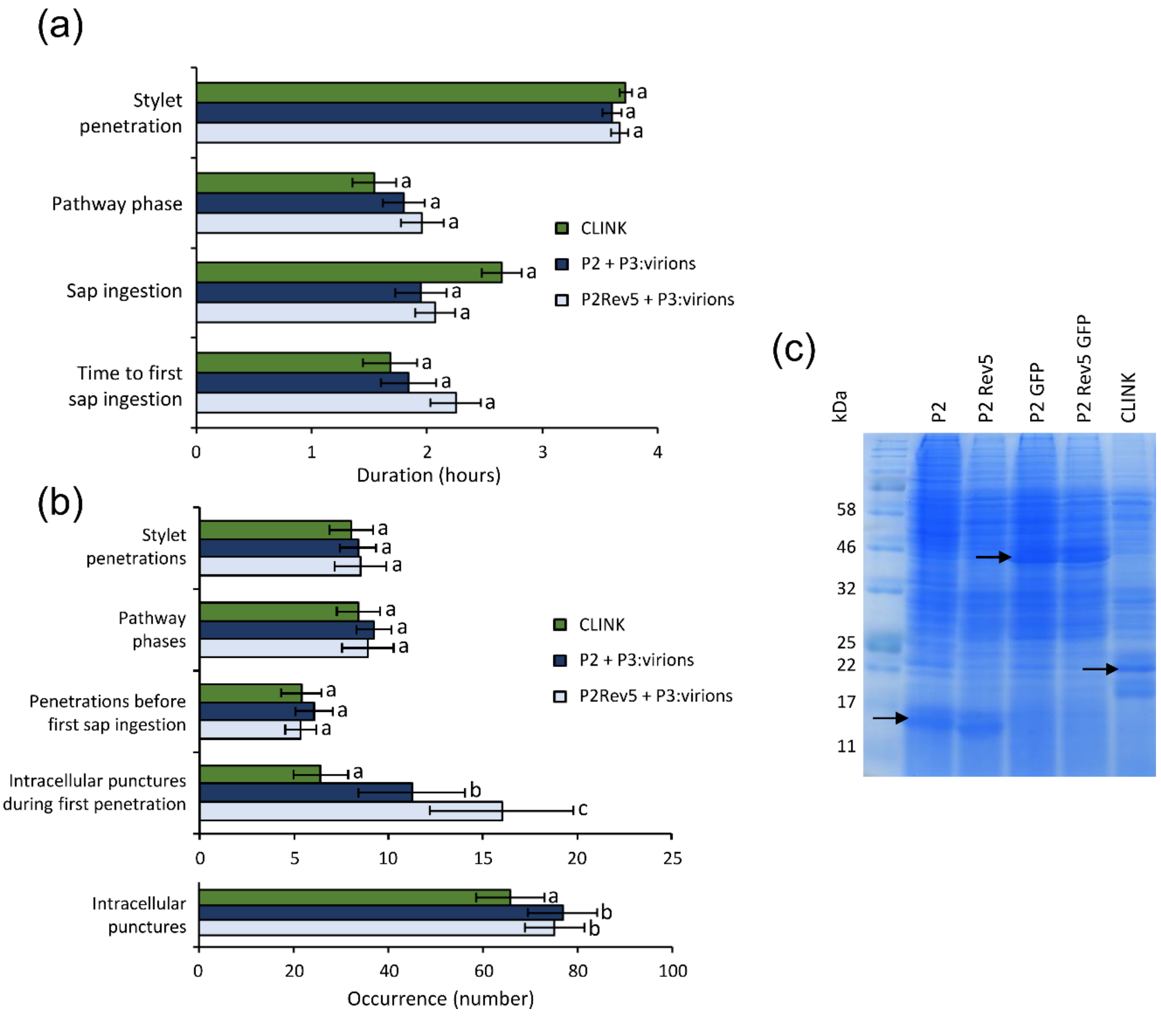

S5 Fig. Effect of recombinant P2Rev5 on aphid behavior. Bars show means and standard errors. (a-b) Feeding behavior of *Myzus persicae* on healthy plants after membrane acquisition of wild type P2 and P3:virions (P2+P3:virions), P2 carrying the mutation Rev5 and P3:virions (P2Rev5+P3:virions) or of an irrelevant protein (CLINK). Before recording aphid feeding behavior on healthy plants, aphids were allowed to feed under EPG control for 1 h on the different artificial media. Only aphids having inserted their stylets for at least 5 min in the artificial media were used for the experiments (N = 29-34). Selected EPG parameters are presented sorted according to (a) duration or (b) occurrence. Different letters show significant differences between treatments as tested by GLM (generalized linear model) followed by pairwise comparisons using "emmeans" ($p < 0.05$ method: Tukey). (a) Statistical analysis of the duration of events revealed no difference for parameters shown (GLM, $p > 0.05$). (b) Statistical analysis of the occurrence of events revealed significant differences for the numbers of intracellular punctures during first penetration and total intracellular punctures (GLM, Df = 2, $\chi 2 = 95.247$, $\chi 2 = 49.683$, $p < 0.001$, respectively). None of the other statistically processed parameters showed a significant difference between treatment (see Supplementary Table 5). (c) Instant Blue stained protein gel after SDS-PAGE of Sf9 crude extracts used for aphid feeding assays on artificial medium. Black arrows point to the position of P2 and P2Rev5, P2:GFP and P2Rev5:GFP, and CLINK, respectively. P2:GFP and P2Rev5:GFP were not used in our experiment.
