# Supplementary material for: The cauliflower mosaic virus transmission helper protein P2 modifies directly the probing behavior of the aphid vector Myzus persicae to facilitate transmission
Source: PLoS Pathog. 2023 Feb 6;19(2):e1011161. doi: 10.1371/journal.ppat.1011161 (PMC9934384; doi:10.1371/journal.ppat.1011161)
Supplement: S6 Fig — The aphid inserted the stylets into tissue after a few seconds (red arrow) and did probing during the rest of the record (red line). In chronological order, behaviors recorded were a pathway phase (dark grey line) with interspersed intracellular test punctures (greens arrows), then salivation into the phloem (medium grey line), and finally a long period of passive phloem sap ingestion (light grey line). (PDF) [file ppat.1011161.s006.pdf]

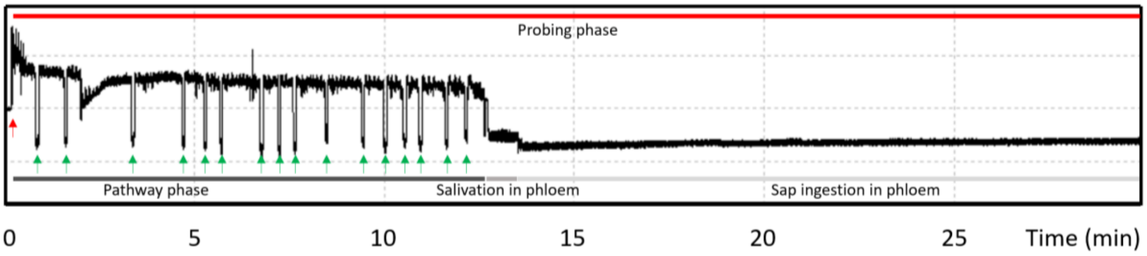


**S6 Fig.** Example of a typical EPG waveform recorded on a leaf. The aphid inserted the stylets into tissue after a few seconds (red arrow) and did probing during the rest of the record (red line). In chronological order, behaviors recorded were a pathway phase (dark grey line) with interspersed intracellular test punctures (greens arrows), then salivation into the phloem (medium grey line), and finally a long period of passive phloem sap ingestion (light grey line).
