# Supplementary material for: The cauliflower mosaic virus transmission helper protein P2 modifies directly the probing behavior of the aphid vector Myzus persicae to facilitate transmission
Source: PLoS Pathog. 2023 Feb 6;19(2):e1011161. doi: 10.1371/journal.ppat.1011161 (PMC9934384; doi:10.1371/journal.ppat.1011161)
Supplement: S7 Fig — At each insertion of the aphid stylets into the artificial medium a signal was observed (red line). Thus, the duration of presence or absence of aphid stylets in the medium could be measured but no behavior phases could be discerned. This might have been due to the high conductivity of the DB5 buffer that was used as a medium. (PDF) [file ppat.1011161.s007.pdf]

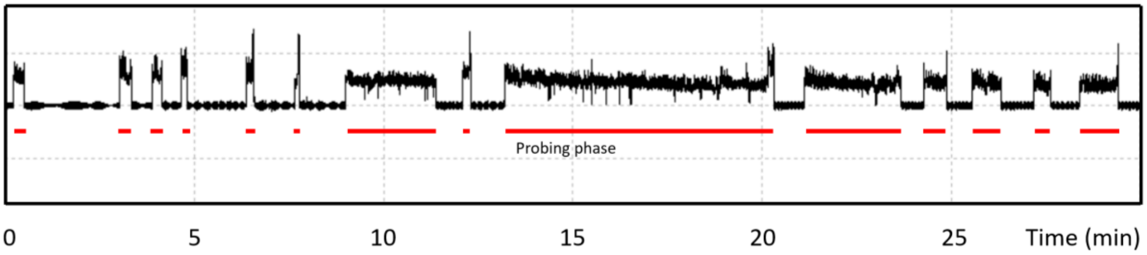

S7 Fig. Typical EPG waveform recorded during membrane feeding. At each insertion of the aphid stylets into the artificial medium a signal was observed (red line). Thus, the duration of presence or absence of aphid stylets in the medium could be measured but no behavior phases could be discerned. This might have been due to the high conductivity of the DB5 buffer that was used as a medium.
