# Supplementary material for: The cauliflower mosaic virus transmission helper protein P2 modifies directly the probing behavior of the aphid vector Myzus persicae to facilitate transmission
Source: PLoS Pathog. 2023 Feb 6;19(2):e1011161. doi: 10.1371/journal.ppat.1011161 (PMC9934384; doi:10.1371/journal.ppat.1011161)
Supplement: S1 Table — (PDF) [file ppat.1011161.s008.pdf]

S1 Table. List of 28 EPG parameters statistically processed for the dataset "Acquisition feeding experiment".

EPG parameters: Acquisition feeding experiment (Fig 1)	Model	Statistiques (Stat, Df, P-value)	Mock (n = 21)		JI (n = 21)		JIΔP2 (n = 24)	
**General probing behaviour (Pr)**								
Number of plant penetrations (n_Pr)	Poisson	**10.756 ; 2 ; <0.001**	15.19 ± 2.13	b	11.95 ± 2.04	a	12.08 ± 2.10	a
Number of brief plant penetrations (< 3 min) (n_bPr)	Poisson	1.797 ; 2 ; 0.407	6.43 ± 1.41		5.43 ± 1.16		5.83 ± 1.09	
Total duration of plant penetrations (s_Pr) (min)	Gamma	3.868 ; 2 ; 0.145	438.96 ± 9.82		459.14 ± 4.19		447.36 ± 6.14	
Time to first plant penetration (t>1Pr) (min)	Cox	4.741 ; 2 ; 0.093	1.81 ± 0.19		1.88 ± 0.38		3.23 ± 0.75	
Duration of the first plant penetration (d_1Pr) (min)	Gamma	0.231 ; 2 ; 0.891	37.40 ± 24.34		24.31± 15.51		25.34 ± 19.77	
Number of plant penetrations before the first sap ingestion in phloem tissues (n_Pr>1E)	Poisson	4.601 ; 2 ; 0.100	6.38 ± 1.21		8.15 ± 2.06		6.96 ± 1.36	
Number of brief plant penetrations before the first sap ingestion in phloem tissues (n_bPr>1E)	Poisson	4.3771 ; 2 ; 0.1121	3.10 ± 0.73		4.15 ± 1.17		4.17 ± 0.84	
**Pathway phase (C)**								
Number of pathway phase (n_C)	Poisson	**24.950 ; 2 ; <0.001**	18.91 ± 2.52	b	13.67 ± 2.10	a	13.62 ± 2.30	a
Total duration of pathway phase (s_C) (min)	Gamma	4.037 ; 2 ; 0.133	196.25 ± 20.77		148.20 ± 23.71		135.61 ± 18.90	
**Feeding behaviour (E: E1 = salivation ; E2 = ingestion)**								
Number of salivation in the phloem tissues (n_E1)	Poisson	**14.712 ; 2 ; <0.001**	3.09 ± 0.43	b	1.38 ± 0.19	a	1.96 ± 0.33	a
Total duration of salivation in the phloem tissues (s_E1) (min)	Gamma	2.337 ; 2 ; 0.311	3.69 ± 0.88		1.81 ± 0.49		2.63 ± 0.93	
Number of sap ingestion in the phloem tissues (n_E2)	Poisson	**6.938 ; 2 ; 0.031**	2.43 ± 0.40	b	1.33 ± 0.19	a	1.75 ± 0.28	ab
Total duration of sap ingestion in the phloem tissues (s_E2) (min)	Gamma	**7.776 ; 2 ; 0.020**	212.69 ± 29.08	b	286.40 ± 26.87	a	280.73 ± 29.11	a
Number of sustained sap ingestion in the phloem tissues (>10 min) (n_sE2)	Poisson	**6.256 ; 2 ; 0.044**	2.19 ± 0.35		1.24 ± 0.15		1.46 ± 0.17	
Total duration of sustained sap ingestion in the phloem tissues (>10 min) (s_sE2) (min)	Gamma	4.132 ; 2 ; 0.127	210.92 ± 29.22		285.88 ± 27.02		279.66 ± 29.27	
Time to first phloem phase (t>1E) (min)	Cox	1.165 ; 2 ; 0.558	131.57 ± 25.61		147.05 ± 26.58		117.82 ± 20.72	
Time to first sap ingestion in the phloem tissues (t>1E2) (min)	Cox	3.373 ; 2 ; 0.185	172.85 ± 30.50		148.08 ± 26.55		121.28 ± 20.86	
**Intracellular puncture (pd)**								
Number of intracellular punctures (n_pd)	Poisson	**37.130 ; 2 ; <0.001**	127.90 ± 16.07	b	110.76 ± 14.61	a	110.13 ± 16.11	a
Total duration of intracellular punctures (s_pd) (min)	Gamma	0.870 ; 2 ; 0.647	10.85 ± 1.33		9.25 ± 1.18		9.39 ± 1.33	
Number of intracellular punctures during the first plant penetration (n_pd/1Pr)	0 inflated	**35.958 ; 2 ; <0.001**	4.95 ± 2.32	b	9.66 ± 4.64	a	3.42 ± 1.31	b
Time to first intracellular puncture (t>1pd) (min)	Cox	2.360 ; 2 ; 0.307	11.63 ± 9.31		2.32 ± 1.06		1.77 ± 0.46	
Number of penetrations before the first intracellular puncture (n_Pr>1pd)	Poisson	3.245 ; 2 ; 0.197	2.23 ± 0.39		1.62 ± 0.32		1.5 ± 0.13	
Number of intracellular punctures per minute of pathway phase (n_pd/minC)	Poisson	3.718 ; 2 ; 0.156	0.72 ± 0.07		0.86 ± 0.06		0.86 ± 0.06	
Average duration of intracellular punctures (a_pd) (sec)	Gamma	0.263 ; 2 ; 0.877	5.22 ± 0.12		5.13 ± 0.17		5.20 ± 0.11	
Median duration of intracellular punctures (m_pd) (sec)	Gamma	0.212 ; 2 ; 0.899	5.10 ± 0.11		5.05 ± 0.15		5.13 ± 0.10	
Duration of the first intracellular puncture (d_1pd) (sec)	Gamma	1.177 ; 2 ; 0.555	5.12 ± 0.33		5.62 ± 0.33		5.35 ± 0.28	
Duration of the second intracellular puncture (d_2pd) (sec)	Gamma	0.234 ; 2 ; 0.890	5.13 ± 0.33		5.65 ± 0.33		5.35 ± 0.28	
Average duration of the first five intracellular punctures (a_pd/1-5pd) (sec)	Gamma	0.300 ; 2 ; 0.861	27.29 ± 1.53		27.41 ± 1.18		26.56 ± 0.92	
