# Supplementary material for: The cauliflower mosaic virus transmission helper protein P2 modifies directly the probing behavior of the aphid vector Myzus persicae to facilitate transmission
Source: PLoS Pathog. 2023 Feb 6;19(2):e1011161. doi: 10.1371/journal.ppat.1011161 (PMC9934384; doi:10.1371/journal.ppat.1011161)
Supplement: S2 Table — (PDF) [file ppat.1011161.s009.pdf]

S2 Table. List of 28 EPG parameters statistically processed for the dataset "inoculation feeding experiment".

EPG parameters: Inoculation feeding experiment (Fig 2)	Model	Statistiques (Stat. Df. P-value)	Mock (n = 26)		JI (n = 22)		JIΔP2 (n = 22)	
**General probing behaviour (Pr)**								
Number of plant penetrations (n_Pr)	Poisson	1.560 ; 2 ; 0.459	9.58 ± 1.72		10.79 ± 4.42		10.39 ± 1.33	
Number of brief plant penetrations (< 3 min) (n_bPr)	Poisson	0.645 ; 2 ; 0.724	4.68 ± 1.02		5.21 ± 0.87		5.11 ± 1.03	
Total duration of plant penetrations (s_Pr) (min)	Gamma	2.710 ; 2 ; 0.258	215.60 ± 4.70		220.19 ± 3.02		223.09 ± 2.06	
Time to first plant penetration (t>1Pr) (min)	Cox	1.758 ; 2 ; 0.415	2.24 ± 0.34		2.99 ± 0.50		2.34 ± 0.27	
Duration of the first plant penetration (d_1Pr) (min)	Gamma	**6.496 ; 2 ; 0.039**	23.82 ± 14.17		21.43 ± 13.37		23.82 ± 23.19	
Number of plant penetrations before the first sap ingestion in phloem tissues (n_Pr>1E)	Poisson	**11.353 ; 2 ; 0.003**	4.47 ± 1.19	a	7.17 ± 1.20	b	6.11 ± 0.87	ab
Number of brief plant penetrations before the first sap ingestion in phloem tissues (n_bPr>1E)	Poisson	**9.274 ; 2 ; 0.010**	2.46 ± 0.62	a	4.3 ± 0.81	b	3.33 ± 0.47	ab
**Pathway phase (C)**								
Number of pathway phase (n_C)	Poisson	2.167 ; 2 ; 0.339	10.58 ± 1.76		12.08 ± 1.52		11.60 ± 1.37	
Total duration of pathway phase (s_C) (min)	Gamma	0.222 ; 2 ; 0.895	97.79 ± 13.95		94.94 ± 10.94		102.02 ± 9.53	
**Feeding behaviour (E: E1 = salivation ; E2 = ingestion)**								
Number of salivation in the phloem tissues (n_E1)	Poisson	0.659 ; 2 ; 0.720	1.26 ± 0.26		1.5 ± 0.21		1.53 ± 0.16	
Total duration of salivation in the phloem tissues (s_E1) (min)	Gamma	2.147 ; 2 ; 0.342	2.34 ± 0.91		1.94 ± 0.45		1.36 ± 0.29	
Number of sap ingestion in the phloem tissues (n_E2)	Poisson	1.767 ; 2 ; 0.413	1.05 ± 0.19		1.33 ± 0.17		1.5 ± 0.17	
Total duration of sap ingestion in the phloem tissues (s_E2) (min)	Gamma	0.587 ; 2 ; 0.756	127.43 ± 19.23		127.63 ± 12.33		115.86 ± 10.75	
Number of sustained sap ingestion in the phloem tissues (>10 min) (n_sE2)	Poisson	1.684 ; 2 ; 0.431	0.95 ± 0.18		1.25 ± 0.17		1.36 ± 0.15	
Total duration of sustained sap ingestion in the phloem tissues (>10 min) (s_sE2) (min)	Gamma	0.748 ; 2 ; 0.688	136.63 ± 18.13		127.24 ± 12.47		119.85 ± 10.23	
Time to first phloem phase (t>1E) (min)	Cox	1.282 ; 2; 0.527	98.45 ± 11.52		87.06 ± 11.52		84.63 ± 10.97	
Time to first sap ingestion in the phloem tissues (t>1E2) (min)	Cox	2.136 2 ; 0.344	109.59 ± 20.43		93.00 ± 13.03		86.21 ± 11.13	
**Intracellular puncture (pd)**								
Number of intracellular punctures (n_pd)	Poisson	**12.629 ; 2 ; <0.001**	83.42 ± 10.59	a	92.71 ± 10.30	b	92.07 ± 8.62	b
Total duration of intracellular punctures (s_pd) (min)	Gamma	0.432 ; 2 ; 0.806	6.71 ± 0.90		7.45 ± 0.84		7.31 ± 0.67	
Number of intracellular punctures during the first plant penetration (n_pd/1Pr)	0 inflated	**39.740 ; 2 ; <0.001**	5.53 ± 2.22	a	3.29 ± 1.31	ab	2.21 ± 0.62	b
Time to first intracellular puncture (t>1pd) (min)	Cox	0.366 ; 2 ; 0.833	0.72 ± 0.19		1.67 ± 0.65		0.94 ± 0.31	
Number of penetrations before the first intracellular puncture (n_Pr>1pd)	Poisson	0.138 ; 2 ; 0.933	1.21 ± 0.10		1.29 ± 0.11		1.18 ± 0.09	
Number of intracellular punctures per minute of pathway phase (n_pd/minC)	Poisson	1.234 ; 2 ; 0.540	1.00 ± 0.09		1.03 ± 0.06		0.94 ± 0.04	
Average duration of intracellular punctures (a_pd) (sec)	Gamma	0.535 ; 2 ; 0.766	4.87 ± 0.11		4.82 ± 0.09		4.78 ± 0.07	
Median duration of intracellular punctures (m_pd) (sec)	Gamma	0.214 ; 2 ; 0.899	4.79 ± 0.11		4.78 ± 0.09		4.73 ± 0.06	
Duration of the first intracellular puncture (d_1pd) (sec)	Gamma	1.416 ; 2 ; 0.493	4.31 ± 0.31		4.81 ± 0.29		4.93 ± 0.27	
Duration of the second intracellular puncture (d_2pd) (sec)	Gamma	1.939 ; 2 ; 0.379	4.82 ± 0.26		5.12 ± 0.34		4.58 ± 0.24	
Average duration of the first five intracellular punctures (a_pd/1-5pd) (sec)	Gamma	0.193 ; 2 ; 0.908	24.82 ± 0.62		24.37 ± 0.93		24.38 ± 0.70	
