# Supplementary material for: The cauliflower mosaic virus transmission helper protein P2 modifies directly the probing behavior of the aphid vector Myzus persicae to facilitate transmission
Source: PLoS Pathog. 2023 Feb 6;19(2):e1011161. doi: 10.1371/journal.ppat.1011161 (PMC9934384; doi:10.1371/journal.ppat.1011161)
Supplement: S3 Table — (PDF) [file ppat.1011161.s010.pdf]

S3 Table. List of 28 EPG parameters statistically processed for the dataset "artificial medium experiment".

EPG parameters: Artificial medium experiment (Fig 3)	Model	Statistiques (Stat, Df, P-value)	Sucrose (n = 26)		DB5 (n = 22)		P3:virions (n = 22)		HP2 (n = 24)		HP2 + P3:virions (n = 21)	
**General probing behaviour (Pr)**												
Number of plant penetrations (n_Pr)	Poisson	**23.228 ; 4 ; <0.001**	10.23 ± 1.49	a	11.27 ± 1.36	a	11.18 ± 1.80	a	11.79 ± 1.50	a	17.95 ± 1.71	b
Number of brief plant penetrations (< 3 min) (n_bPr)	Poisson	**28.823 ; 4 ; <0.001**	4.12 ± 0.87	a	5.27 ± 0.96	a	4.86 ± 1.19	a	4.66 ± 0.83	a	7.62 ± 1.49	b
Total duration of plant penetrations (s_Pr) (min)	Gamma	3.432 ; 4 ; 0.488	219.46 ± 3.69		215.53 ± 4.11		224.37 ± 2.74		219.65 ± 3.22		217.85 ± 2.91	
Time to first plant penetration (t>1Pr) (min)	Cox	4.067 ; 4 ; 0.397	1.84 ± 0.34		2.88 ± 0.86		1.57 ± 0.26		1.80 ± 0.31		1.67 ± 0.32	
Duration of the first plant penetration (d_1Pr) (min)	Gamma	7.455 ; 4 ; 0.114	4.34 ± 0.20		4.33 ± 0.35		4.41 ± 0.25		4.23 ± 0.29		4.63 ± 0.21	
Number of plant penetrations before the first sap ingestion in phloem tissues (n_Pr>1E)	Poisson	**20.090 ; 4 ; <0.001**	5.14 ± 1.00	a	7.26 ± 1.15	ab	6.47 ± 1.75	ab	7.28 ± 1.64	ab	8.74 ± 1.59	b
Number of brief plant penetrations before the first sap ingestion in phloem tissues (n_bPr>1E)	Poisson	**19.636 ; 4 ; <0.001**	2.52 ± 0.71	a	4.05 ± 1.00	ab	3.84 ± 1.15	ab	3.39 ± 0.73	ab	5.16 ± 1.25	b
**Pathway phase (C)**												
Number of pathway phase (n_C)	Poisson	**21.336 ; 4 ; <0.001**	11.42 ± 1.49	a	12.5 ± 1.52	a	12.27 ± 1.87	a	13.13 ± 1.54	ab	16.14 ± 1.86	b
Total duration of pathway phase (s_C) (min)	Gamma	1.515 ; 4 ; 0.824	109.75 ± 12.10		118.6 ± 12.31		125.19 ± 14.16		129.15 ± 12.30		124.22 ± 12.20	
**Feeding behaviour (E: E1 = salivation ; E2 = ingestion)**												
Number of salivation in the phloem tissues (n_E1)	Poisson	0.571 ; 4 ; 0.966	1.35 ± 0.25		1.5 ± 0.34		1.55 ± 0.29		1.58 ± 0.26		1.52 ± 0.31	
Total duration of salivation in the phloem tissues (s_E1) (min)	Gamma	0.292 ; 4 ; 0.990	2.34 ± 0.98		2.66 ± 1.10		2.90 ± 1.11		2.99 ± 0.67		2.53 ± 0.84	
Number of sap ingestion in the phloem tissues (n_E2)	Poisson	0.306 ; 4 ; 0.989	1.23 ± 0.24		1.27 ± 0.22		1.32 ± 0.24		1.36 ± 0.40		1.38 ± 0.31	
Total duration of sap ingestion in the phloem tissues (s_E2) (min)	Gamma	2.091 ; 4 ; 0.719	124.51 ± 13.74		102.6 ± 15.79		114.32 ± 15.46		106.96 ± 15.15		96.85 ± 14.56	
Number of sustained sap ingestion in the phloem tissues (>10 min) (n_sE2)	Poisson	1.240 ; 4 ; 0.872	1.12 ± 0.17		0.91 ± 0.13		1.09 ± 0.16		0.88 ± 0.15		1.10 ± 0.15	
Total duration of sustained sap ingestion in the phloem tissues (>10 min) (s_sE2) (min)	Gamma	0.766 ; 4 ; 0.943	123.73 ± 13.72		112.65 ± 15.80		112.58 ± 15.70		117.54 ± 13.73		107.05 ± 14.17	
Time to first phloem phase (t>1E) (min)	Cox	1.579 ; 4 ; 0.813	113.24 ± 15.53		112.30 ± 13.70		102.03 ± 16.64		118.93 ± 18.41		99.95 ± 15.25	
Time to first sap ingestion in the phloem tissues (t>1E2) (min)	Cox	1.470 ; 4 ; 0.832	114.12 ± 15.47		113.04 ± 13.65		108.16 ± 16.61		124.51 ± 17.57		111.08 ± 16.49	
**Intracellular puncture (pd)**												
Number of intracellular punctures (n_pd)	Poisson	**72.429 ; 4 ; <0.001**	80.42 ± 8.98	a	88.55 ± 9.86	b	93.23 ± 10.62	bc	96.63 ± 8.99	cd	102.48 ± 10.26	d
Total duration of intracellular punctures (s_pd) (min)	Gamma	2.041 ; 4 ; 0.728	6.69 ± 0.80		7.05 ± 0.80		7.89 ± 0.88		7.51 ± 0.67		8.03 ± 0.83	
Number of intracellular punctures during the first plant penetration (n_pd/1Pr)	0 inflated	**72.430 ; 4 ; <0.001**	7.65 ± 2.95	b	3.45 ± 1.41	a	6.31 ± 2.89	b	11.17 ± 4.8	c	5.19 ± 2.26	ab
Time to first intracellular puncture (t>1pd) (min)	Cox	1.185 ; 4 ; 0.881	3.40 ± 2.00		5.35 ± 2.91		1.00 ± 0.28		1.11 ± 0.37		10.0 ± 0.32	
Number of penetrations before the first intracellular puncture (n_Pr>1pd)	Poisson	**18.061 ; 4 ; <0.001**	1.27 ± 0.16	a	2.55 ± 0.93	b	1.37 ± 0.07	a	1.29 ± 0.20	a	1.24 ± 1.12	a
Number of intracellular punctures per minute of pathway phase (n_pd/minC)	Poisson	1.703 ; 4 ; 0.790	0.85 ± 0.06		0.81 ± 0.07		0.84 ± 0.07		0.83 ± 0.07		0.93 ± 0.08	
Average duration of intracellular punctures (a_pd) (sec)	Gamma	**13.834 ; 4 ; 0.008**	4.89 ± 0.07	a	4.77 ± 0.10	ab	5.12 ± 0.11	ab	4.67 ± 0.11	b	4.68 ± 0.08	b
Median duration of intracellular punctures (m_pd) (sec)	Gamma	**10.107 ; 4 ; 0.039**	4.80 ± 0.09	a	4.68 ± 0.10	ab	4.98 ± 0.09	ab	4.61 ± 0.11	ab	4.66 ± 0.08	b
Duration of the first intracellular puncture (d_1pd) (sec)	Gamma	1.202 ; 4 ; 0.878	4.34 ± 0.20		4.33 ± 0.35		4.41 ± 0.25		4.26 ± 0.29		4.63 ± 0.21	
Duration of the second intracellular puncture (d_2pd) (sec)	Gamma	1.069 ; 4 ; 0.899	4.67 ± 0.23		4.59 ± 0.33		4.92 ± 0.29		4.81 ± 0.29		4.56 ± 0.31	
Average duration of the first five intracellular punctures (a_pd/1-5pd) (sec)	Gamma	2.241 ; 4 ; 0.692	23.20 ± 0.58		25.14 ± 1.66		24.06 ± 0.71		24.03 ± 0.89		23.49 ± 0.71	
