# Supplementary material for: The cauliflower mosaic virus transmission helper protein P2 modifies directly the probing behavior of the aphid vector Myzus persicae to facilitate transmission
Source: PLoS Pathog. 2023 Feb 6;19(2):e1011161. doi: 10.1371/journal.ppat.1011161 (PMC9934384; doi:10.1371/journal.ppat.1011161)
Supplement: S4 Table — (PDF) [file ppat.1011161.s011.pdf]

S4 Table. List of 28 EPG parameters statistically processed for the dataset “JI-P2Rev5 experiment”.

EPG parameters: JI-P2Rev5 experiment (Fig 4)	Model	Statistiques (Stat, Df, P-value)	Mock (n = 27)		JI (n = 28)		JI-P2Rev5 (n = 26)	
**General probing behaviour (Pr)**								
Number of plant penetrations (n_Pr)	Poisson	5.673 ; 2 ; 0.059	14.37 ± 2.17		12.11 ± 1.60		13.73 ± 2.09	
Number of brief plant penetrations (< 3 min) (n_bPr)	Poisson	**19.837 ; 2 ; <0.001**	10.15 ± 1.78	b	6.68 ± 0.94	a	8.34 ± 1.62	ab
Total duration of plant penetrations (s_Pr) (min)	Gamma	1.146 ; 2 ; 0.564	209.82 ± 4.68		215.48 ± 3.80		215.29 ± 4.28	
Time to first plant penetration (t>1Pr) (min)	Cox	**9.116 ; 2 ; 0.010**	2.20 ± 0.46	ab	3.00 ± 0.46	a	1.55 ± 0.31	b
Duration of the first plant penetration (d_1Pr) (min)	Gamma	**19.838 ; 2 ; <0.001**	2.66 ± 1.65	a	27.96 ± 13.90	a	0.98 ± 0.43	a
Number of plant penetrations before the first sap ingestion in phloem tissues (n_Pr>1E)	Poisson	**13.709 ; 2 ; 0.001**	11 ± 2.03	b	7.79 ± 1.57	a	9.48 ± 1.55	ab
Number of brief plant penetrations before the first sap ingestion in phloem tissues (n_bPr>1E)	Poisson	**24.266 ; 2 ; <0.001**	9.08 ± 1.65	b	5.38 ± 1.02	a	6.88 ± 1.31	a
**Pathway phase (C)**								
Number of pathway phase (n_C)	Poisson	5.452 ; 2 ; 0.065	15.78 ± 2.30		13.43 ± 1.63		15.04 ± 2.22	
Total duration of pathway phase (s_C) (min)	Gamma	0.455 ; 2 ; 0.797	75.85 ± 9.71		84.88 ± 11.28		83.87 ± 10.51	
**Feeding behaviour (E: E1 = salivation ; E2 = ingestion)**								
Number of salivation in the phloem tissues (n_E1)	Poisson	0.259 ; 2 ; 0.879	2 ± 0.26		1.89 ± 0.32		1.81 ± 0.23	
Total duration of salivation in the phloem tissues (s_E1) (min)	Gamma	0.301 ; 2 ; 0.861	1.62 ± 0.36		1.66 ± 0.30		1.43 ± 0.29	
Number of sap ingestion in the phloem tissues (n_E2)	Poisson	0.296 ; 2 ; 0.862	1.93 ± 0.26		1.79 ± 0.30		1.73 ± 0.22	
Total duration of sap ingestion in the phloem tissues (s_E2) (min)	Gamma	0.198 ; 2 ; 0.906	136.31 ± 10.04		140.28 ± 14.00		132.20 ± 13.26	
Number of sustained sap ingestion in the phloem tissues (>10 min) (n_sE2)	Poisson	1.697 ; 2 ; 0.428	1.52 ± 0.22		1.18 ± 0.15		1.15 ± 0.11	
Total duration of sustained sap ingestion in the phloem tissues (>10 min) (s_sE2) (min)	Gamma	0.097 ; 2 ; 0.952	140.64 ± 10.05		137.04 ± 14.22		135.17 ± 13.13	
Time to first phloem phase (t>1E) (min)	Cox	0.894 ; 2 ; 0.640	75.69 ± 10.72		90.34 ± 15.75		73.71 ± 10.92	
Time to first sap ingestion in the phloem tissues (t>1E2) (min)	Cox	0.801 ; 2 ; 0.67	77.93 ± 11.30		91.16 ± 15.71		75.81 ± 11.43	
**Intracellular puncture (pd)**								
Number of intracellular punctures (n_pd)	Poisson	**23.692 ; 2 ; <0.001**	83.59 ± 9.03	a	84.71 ± 9.54	a	95.04 ± 8.94	b
Total duration of intracellular punctures (s_pd) (min)	Gamma	1.844 ; 2 ; 0.398	5.95 ± 0.65		6.08 ± 0.70		7.40 ± 1.12	
Number of intracellular punctures during the first plant penetration (n_pd/1Pr)	0 inflated	**77.32 ; 2 ; <0.001**	2.22 ± 1.16	a	5.93 ± 2.38	b	1.35 ± 0.36	a
Time to first intracellular puncture (t>1pd) (min)	Cox	0.759 ; 2 ; 0.684	0.81 ± 0.30		0.30 ± 0.10		0.91 ± 0.41	
Number of penetrations before the first intracellular puncture (n_Pr>1pd)	Poisson	1.069 ; 2 ; 0.586	1.30 ± 0.14		1 ± 0		1.12 ± 0.06	
Number of intracellular punctures per minute of pathway phase (n_pd/minC)	Poisson	0.533 ; 2 ; 0.766	1.22 ± 0.07		1.21 ± 0.08		1.28 ± 0.06	
Average duration of intracellular punctures (a_pd) (sec)	Gamma	0.863 ; 2 ; 0.649	4.26 ± 0.07		4.29 ± 0.06		4.43 ± 0.23	
Median duration of intracellular punctures (m_pd) (sec)	Gamma	0.747 ; 2 ; 0.688	4.20 ± 0.07		4.25 ± 0.05		4.19 ± 0.05	
Duration of the first intracellular puncture (d_1pd) (sec)	Gamma	2.130 ; 2 ; 0.345	4.26 ± 0.24		4.73 ± 0.25		4.51 ± 0.20	
Duration of the second intracellular puncture (d_2pd) (sec)	Gamma	0.977 ; 2 ; 0.614	4.45 ± 0.28		4.16 ± 0.19		4.43 ± 0.22	
Average duration of the first five intracellular punctures (a_pd/1-5pd) (sec)	Gamma	0.737 ; 2 ; 0.692	21.67 ± 0.75		22.15 ± 0.46		22.40 ± 0.58	
