# Supplementary material for: The cauliflower mosaic virus transmission helper protein P2 modifies directly the probing behavior of the aphid vector Myzus persicae to facilitate transmission
Source: PLoS Pathog. 2023 Feb 6;19(2):e1011161. doi: 10.1371/journal.ppat.1011161 (PMC9934384; doi:10.1371/journal.ppat.1011161)
Supplement: S5 Table — (PDF) [file ppat.1011161.s012.pdf]

S5 Table. List of 28 EPG parameters statistically processed for the dataset “recombinant P2Rev5 experiment”.

**EPG parameters: P2Rev5 experiment (S5 Fig)**	**Model**	**Statistiques (Stat, Df, P-value)**	**CLINK (n = 34)**		**P2 + P3:virions (n = 29)**		**P2Rev5 + P3:virions (n = 34)**	
**General probing behaviour (Pr)**								
Number of plant penetrations (n_Pr)	Poisson	2.136 ; 2 ; 0.344	8.03 ± 1.14		8.41 ± 0.96		8.53 ± 1.38	
Number of brief plant penetrations (< 3 min) (n_bPr)	Poisson	2.828 ; 2 ; 0.244	2.97 ± 0.65		2.72 ± 0.49		3.24 ± 0.94	
Total duration of plant penetrations (s_Pr) (min)	Gamma	0.536 ; 2 ; 0.769	223.35 ± 3.19		216.21 ± 5.11		220.27 ± 4.39	
Time to first plant penetration (t>1Pr) (min)	Cox	2.255 ; 2 ; 0.324	3.41 ± 1.62		4.30 ± 2.97		1.50 ± 0.24	
Duration of the first plant penetration (d_1Pr) (min)	Gamma	0.704 ; 2 ; 0.703	35.17 ± 12.64		25.70 ± 8.74		40.12 ± 11.96	
Number of plant penetrations before the first sap ingestion in phloem tissues (n_Pr>1E)	Poisson	3.525 ; 2 ; 0.172	5.38 ± 1.08		6.08 ± 0.99		5.33 ± 0.82	
Number of brief plant penetrations before the first sap ingestion in phloem tissues (n_bPr>1E)	Poisson	0.537 ; 2 ; 0.765	2.62 ± 0.73		2.28 ± 0.52		2.04 ± 0.44	
**Pathway phase (C)**								
Number of pathway phase (n_C)	Poisson	2.437 ; 2 ; 0.296	8.41 ± 1.17		9.24 ± 0.92		8.91 ± 1.37	
Total duration of pathway phase (s_C) (min)	Gamma	3.145 ; 2 ; 0.208	92.71 ± 11.50		108.05 ± 10.81		117.48 ± 11.11	
**Feeding behaviour (E: E1 = salivation ; E2 = ingestion)**								
Number of salivation in the phloem tissues (n_E1)	Poisson	1.089 ; 2 ; 0.580	1.03 ± 0.14		1.28 ± 0.22		1 ± 0.13	
Total duration of salivation in the phloem tissues (s_E1) (min)	Gamma	1.781 ; 2 ; 0.410	2.36 ± 0.97		1.70 ± 0.42		1.25 ± 0.14	
Number of sap ingestion in the phloem tissues (n_E2)	Poisson	1.425 ; 2 ; 0.490	0.91 ± 0.11		1.21 ± 0.18		0.97 ± 0.13	
Total duration of sap ingestion in the phloem tissues (s_E2) (min)	Gamma	**6.279 ; 2 ; 0.043**	159.03 ± 10.39	a	117.01 ± 13.31	a	124.25 ± 10.43	a
Number of sustained sap ingestion in the phloem tissues (>10 min) (n_sE2)	Poisson	0.561 ; 2 ; 0.755	0.88 ± 0.09		1.07 ± 0.16		0.97 ± 0.13	
Total duration of sustained sap ingestion in the phloem tissues (>10 min) (s_sE2) (min)	Gamma	5.695 ; 2 ; 0.058	158.72 ± 10.51		120.84 ± 13.16		124.25 ± 10.43	
Time to first phloem phase (t>1E) (min)	Cox	2.567 ; 2 ; 0.277	96.23 ± 13.32		108.96 ± 14.37		133.24 ± 13.07	
Time to first sap ingestion in the phloem tissues (t>1E2) (min)	Cox	2.090 ; 2 ; 0.352	100.93 ± 14.11		110.32 ± 14.21		134.93 ± 12.96	
**Intracellular puncture (pd)**								
Number of intracellular punctures (n_pd)	Poisson	**49.683 ; 2 ; <0.001**	65.71 ± 7.23	a	76.79 ± 7.35	b	75.06 ± 6.31	b
Total duration of intracellular punctures (s_pd) (min)	Gamma	3.087 ; 2 ; 0.214	4.90 ± 0.53		5.89 ± 0.53		5.86 ± 0.49	
Number of intracellular punctures during the first plant penetration (n_pd/1Pr)	0 inflated	**95.247 ; 2 ; <0.001**	6.41 ± 1.47	a	11.24 ± 2.82	b	16 ± 3.82	c
Time to first intracellular puncture (t>1pd) (min)	Cox	0.184 ; 2 ; 0.912	1.26 ± 0.38		1.27 ± 0.48		1.68 ± 0.62	
Number of penetrations before the first intracellular puncture (n_Pr>1pd)	Poisson	0.052 ; 2 ; 0.974	1.18 ± 0.07		1.14 ± 0.08		1.09 ± 0.06	
Number of intracellular punctures per minute of pathway phase (n_pd/minC)	Poisson	3.589 ; 2 ; 0.166	0.91 ± 0.07		0.77 ± 0.04		0.76 ± 0.05	
Average duration of intracellular punctures (a_pd) (sec)	Gamma	2.146 ; 2 ; 0.342	4.50 ± 0.08		4.64 ± 0.07		4.68 ± 0.05	
Median duration of intracellular punctures (m_pd) (sec)	Gamma	1.015 ; 2 ; 0.602	4.48 ± 0.08		4.58 ± 0.06		4.64 ± 0.05	
Duration of the first intracellular puncture (d_1pd) (sec)	Gamma	1.634 ; 2 ; 0.442	4.52 ± 0.18		5.15 ± 1.00		4.40 ± 0.18	
Duration of the second intracellular puncture (d_2pd) (sec)	Gamma	2.795 ; 2 ; 0.247	4.45 ± 0.16		4.84 ± 0.20		4.74 ± 0.24	
Average duration of the first five intracellular punctures (a_pd/1-5pd) (sec)	Gamma	2.866 ; 2 ; 0.239	22.46 ± 0.57		24.02 ± 1.12		22.65 ± 0.50	
