# Supplementary material for: The cauliflower mosaic virus transmission helper protein P2 modifies directly the probing behavior of the aphid vector Myzus persicae to facilitate transmission
Source: PLoS Pathog. 2023 Feb 6;19(2):e1011161. doi: 10.1371/journal.ppat.1011161 (PMC9934384; doi:10.1371/journal.ppat.1011161)
Supplement: S6 Table — (PDF) [file ppat.1011161.s013.pdf]

S6 Table. List of p-values of different pairwise comparisons performed when a significant effect was detected with GLM (p-value adjustment with Tukey method at the 0.05 significance level).

Fig 1c

Comparisons	Stylet penetration	Pathway phase	sap ingestion	Time to first sap ingestion
Mock - JI	NA	NA	**0.0283**	NA
Mock - JIΔP2	NA	NA	**0.0323**	NA
JI- JIΔP2	NA	NA	0.9774	NA

Fig 1d

Comparisons	stylet penetrations	Pathway phases	Sap ingestions	Penetrations before first sap ingestion	Intracellular punctures during first penetration	Intracellular punctures
Mock - JI	**0.0125**	**0.0001**	**0.0291**	NA	**0.0361**	**<.0001**
Mock - JIΔP2	**0.0133**	**<.0001**	0.2575	NA	0.5661	**<.0001**
JI- JIΔP2	0.9912	0.9992	0.5050	NA	**0.0001**	0.9776

Fig 2a

Comparisons	Stylet penetration	Pathway phase	sap ingestion	Time to first sap ingestion
Mock - JI	NA	NA	NA	NA
Mock - JIΔP2	NA	NA	NA	NA
JI- JIΔP2	NA	NA	NA	NA

Fig 2b

Comparisons	stylet penetrations	Pathway phases	Penetrations before first sap ingestion	Intracellular punctures during first penetration	Intracellular punctures
Mock - JI	NA	NA	**0.0031**	0.0650	**0.0038**
Mock - JIΔP2	NA	NA	0.0775	**0.0007**	**0.0057**
JI- JIΔP2	NA	NA	0.3121	0.1778	0.9692

Fig 3

Comparisons	stylet penetrations	Pathway phases	Penetrations before first sap ingestion	Intracellular punctures during first penetration	Intracellular punctures
P3:virions - Sucrose	0.8532	0.9137	0.4061	0.7614	**<.0001**
DB5 - Sucrose	0.8073	0.8187	0.0559	**0.0004**	**0.0191**
DB5 - P3:virions	1.0000	0.9995	0.8862	**0.0244**	0.4789
HP2 - Sucrose	0.4573	0.4233	0.0583	**0.0481**	**<.0001**
HP2 - P3:virions	0.9737	0.9278	0.8843	**0.0008**	0.7623
HP2 - DB5	0.9856	0.9764	1.0000	**<.0001**	**0.0363**
HP2 + P3:virions - Sucrose	**0.0001**	**0.0001**	**0.0002**	0.1784	**<.0001**
HP2 + P3:virions - P3:virions	**0.0058**	**0.0070**	0.0862	0.8240	**0.0185**
HP2 + P3:virions - DB5	**0.0079**	**0.0141**	0.4950	0.3563	**<.0001**
HP2 + P3:virions - HP2	**0.0309**	0.0626	0.5210	**<.0001**	0.2836

Fig 4c

Comparisons	stylet penetrations	Pathway phases	Penetrations before first sap ingestion	Intracellular punctures during first penetration	Intracellular punctures
Mock - JI	NA	NA	**0.0007**	**<.0001**	0.8929
Mock - JI-P2-Rev5	NA	NA	0.2078	0.0697	**<.0001**
JI - JI-P2-Rev5	NA	NA	0.1109	**<.0001**	**0.0002**
