# Supplementary material for: The cauliflower mosaic virus transmission helper protein P2 modifies directly the probing behavior of the aphid vector Myzus persicae to facilitate transmission
Source: PLoS Pathog. 2023 Feb 6;19(2):e1011161. doi: 10.1371/journal.ppat.1011161 (PMC9934384; doi:10.1371/journal.ppat.1011161)
Supplement: S1 Text — (PDF) [file ppat.1011161.s014.pdf]

S1 Text. Production of recombinant P2Rev5 and CLINK.

We used previously published recombinant baculoviruses expressing P2 [1] or P2Rev5 [2] from the late p10 promoter. As a negative control, we used a recombinant baculovirus expressing the faba bean necrotic stunt virus (FBNSV) protein CLINK under control of the p10 promoter. CLINK has no homology to any CaMV protein. It interacts intracellularly with a retinoblastoma-related protein and interferes with the cell cycle [3]. Therefore, it should not have any effect when acquired by aphids orally. The CLINK coding sequence was inserted into the baculovirus genome by Agate Bioservices (Bagard, France) using the BacTen system [4].

For protein production, *Sf9* cells were infected with the recombinant baculoviruses and incubated for 2 days at 28 °C. Cells were harvested by centrifugation for 5 min at 500 g and frozen at -80 °C. Then cells were thawed and resuspended in DB5 buffer (50 mM HEPES pH 8.0, 500 mM Li2SO4, 0.5 mM EGTA, 0.2 % CHAPS) supplemented with SigmaFast Protease Inhibitor (EDTA-free). The suspension was clarified by centrifugation for 10 min at 10,000 g, the supernatant divided in aliquots and frozen at -80 °C until use. Recombinant protein expression was verified by Instant Blue staining of protein gels after SDS-PAGE (S5c Fig).

References

1 Blanc, S., M .Cerutti, M. Usmany, J. M. Vlak, and R. Hull. 1993. “Biological Activity of Cauliflower Mosaic Virus Aphid Transmission Factor Expressed in a Heterologous System.” Virology 192 (2): 643–50. https://doi.org/10.1006/viro.1993.1080.

2 Moreno, A., E. Hébrard, M. Uzest, S. Blanc, and A. Fereres. 2005. “A Single Amino Acid Position in the Helper Component of Cauliflower Mosaic Virus Can Change the Spectrum of Transmitting Vector Species.” Journal of Virology 79 (21): 13587–93. https://doi.org/10.1128/JVI.79.21.13587-13593.2005.

3 Lageix, S., O. Catrice, J.-M. Deragon, B. Gronenborn, T. Pélissier, and B.C. Ramírez. 2007. “The Nanovirus-Encoded Clink Protein Affects Plant Cell Cycle Regulation through Interaction with the Retinoblastoma-Related Protein.” Journal of Virology 81 (8): 4177–85. https://doi.org/10.1128/JVI.02103-06.

4 Chaabihi, H., C. Cêtre, and A. Berne. 1997. “A New Vector for Efficient Generation of P10-Single-Late-Promoter Recombinant Baculoviruses.” Journal of Virological Methods 63 (1–2): 1–7. https://doi.org/10.1016/s0166-0934(96)02104-0.
