# Supplementary material for: The cauliflower mosaic virus transmission helper protein P2 modifies directly the probing behavior of the aphid vector Myzus persicae to facilitate transmission
Source: PLoS Pathog. 2023 Feb 6;19(2):e1011161. doi: 10.1371/journal.ppat.1011161 (PMC9934384; doi:10.1371/journal.ppat.1011161)
Supplement: Attachment — Submitted filename: Responses-to-Reviewers.pdf [file ppat.1011161.s016.pdf]

Thanks to the reviewers for the enormous time and effort spent on our paper. We highly appreciate your extremely constructive scientific and linguistic comments and suggestions. Please find our responses interspersed with your comments.

Besides the demanded changes, the first part of the discussion was entirely revised to increase clarity and to shorten it. We also introduced, as for the other sections, subtitles to the discussion. Finally, for editing purposes, the significance statement was changed to an Author Summary, and the Supplementary Information transferred to a proper file.

Reviewer's Responses to Questions

Part I - Summary

Please use this section to discuss strengths/weaknesses of study, novelty/significance, general execution and scholarship.

Reviewer #1: This is a well researched and written manuscript. The authors provide evidence that the helper component (P2 protein) of a plant virus (CaMV) can directly modify the probing behaviour of its aphid vector. The authors make use of electrical penetration graph (EPG), wild type virus and a mutant form that doesn't produce the P2 protein to show that aphid probing and feeding actions associated with virus acquisition and successful inoculation are enhanced in the presence of P2 protein. These findings are significant in that they demonstrate that initial contact between an aphid vector and the virus results in behavioural changes in the aphid and that these changes can be caused by the direct binding of P2 to receptors in the aphid stylet. Further, the authors demonstrate the power of EPG as a valuable tool in deciphering how viruses manipulate the behaviour of their insect vectors. These results are important in modelling acquisition and dispersal of viruses by aphid vectors. These findings will be valuable to the scholarship of molecular plant virology and plant-virus-insect interactions whose significance will increase as novel ways to control aphids and the viruses they spread are sought.

Reviewer #2: In regard of novelty, the study addresses a less well studied area where the authors investigated if a virus factor can modify vector fitness or behaviour. They chose CaMV P2 protein and Myzus persicae or the green peach aphid as CaMV vector. CaMV is a semi-persistently transmitted plant virus whereby it is not known if CaMV can have a direct involvement in modifying vector (aphid) behaviour, and its implication on onward transmission of the virus.
The strength of the study lies in the structured way they conducted their studies.

The group did prior work on CaMV , i.e.: Chesnais et al., 2020, 2021; Drucker et al., 2002 as referenced in the manuscripts. They discovered that P6-TAV, a multifunctional protein of CaMV, led to altered feeding behaviour of the aphids on Arabidopsis and this is likely to be mediated by indirect host-mediated effect as P6-TAV is involved mainly in modification of host plant physiology. CaMV P2 protein is a helper protein which has been shown to form a protein bridge between the aphid's stylet and the virions. Hence the authors chose to investigate P2 protein in this study as a candidate for a virus-encoded factor which may modify vector behaviour.

Overall this paper is well laid out, where the authors investigated whether P2 protein of CaMV

are involved in modifying aphid feeding behaviour as CaMV vector. The authors utilised wild-type CaMV virus (JI), mutant virus with abolished P2 (JIdP2), and a mutant virus whereby P2 has lost the ability to bind to the aphid mouthparts (JI-P2Rev5). The authors further tested whether the effect of P2 on aphid feeding behaviour is independent from other plant factors by using membrane feeding set up to allow aphids to feed directly on food solution with P2 virion, or P2:P3 virion complexes. The results did add new knowledge in the field.

The weakness of this study lies in the sentence structure and language, as well as there are additional experiments which can help clarify the results. There were several occasions where the choice of word and sentence structure need editing. The titles for the subsection in the results part of the manuscript also needs re-phrasing to increase its accuracy in reflecting the experimental result, and to avoid over assumption. Several statements are seemingly contradictory to one another, which can be due to poor sentence structuring and will need to be re-written to improve its clarity. The labelling on Figure 1 needs to be corrected (further details below). The suggested experiments are described in the following section.

Thanks for the many language corrections in Part III.

Reviewer #3: This manuscript presents convincing evidence for a role of CaMV P2 in influencing aphid vector behaviour directly. The implication that a protein of a non-circulative virus manipulates the vector is highly significant and will be of broad interest to researchers in plant-pathogen-vector interactions. The experimental approaches are rigorous and nicely combine EPG recording with use of mutant viruses and an artificial membrane feeding system to provide consistent and significant results. The findings are novel and the work is suitable for publication following some minor changes.

Part II – Major Issues: Key Experiments Required for Acceptance
Please use this section to detail the key new experiments or modifications of existing experiments that should be absolutely required to validate study conclusions.

Generally, there should be no more than 3 such required experiments or major modifications for a "Major Revision" recommendation. If more than 3 experiments are necessary to validate the study conclusions, then you are encouraged to recommend "Reject".

Reviewer #1: n/a

Reviewer #2: Fig 1b : can the authors check if the labelling is correct on the westen blot? If anti P2 is an antibody specific for P2 protein, then why does bands appear on JIΔP2 samples whereby P2 were not supposed to be present? Please verify.

Thank you for noting the inverted label on Figure 1b. We corrected the labeling.

Have the authors tested whether the aphids were able to acquire the mutant CaMV JI-P2Rev5 virus?

We did not formally test the acquisition of the mutant CaMV JI-P2Rev5, with plant-to-plant transmission tests as a previous study already showed that a CaMV isolate harboring the same P2Rev5 mutation is not transmissible (Moreno et al., 2005, doi:

10.1128/jvi.79.21.13587-13593.2005). Furthermore, we did not test formally, using membrane feeding assays, whether aphids can acquire and transmit CaMV JI-P2Rev5 virus particles when wild type P2 is added to the feeding medium. However, since the mutant codes for wild type coat protein and P3, CaMV JI-Rev5 particles should be transmitted under this condition, just as non-transmissible CaMV mutants CM1841 and C4-184 are when aphids had previous access to wild type P2 (Blanc et al. 1993, doi:10.1006/viro.1993.1080; Khelifa et al. 2007, doi:10.1099/vir.0.83009-0).

The authors did demonstrate that turnip plants were infected with CaMV JI-P2Rev5 virus, but with P2 being unable to bind to the aphid stylet – have the authors checked whether aphids can acquire and subsequently transmit CaMV JI-P2Rev5 mutant virus?

Please see our response to your previous comment.

Figure 4 did show that aphids fed on JI-infected plants performed significantly more intracellular punctures than aphids fed on CaMV JI-P2Rev5 infected plants. Whilst Figure S4 showed no significant difference between aphid feeding behaviour when different parameters were measured than Fig 4 (sap ingestion and time to first ingestion) using EPG on JI-infected and JI-P2Rev5 plants.

Figure 4 shows occurrence of different feeding behaviors, while Figure S4 shows duration of feeding behaviors. We observed significant differences only for occurrence, not for total duration.

For L. 230 (Figure 3) : Did the authors detect P2 and/or P3:virions titer or verify its presence in the individual aphids after they were fed on the artificial membrane containing P2 and P3:virions? this is to verify that the effects observed on the aphids were genuinely due to the P2 and/or P3 being taken up by the aphids and not just due to aphids experiencing feeding difficulties on the membrane for example? . Figure S2 showed viral components used for aphid feeding assay on the artificial medium, but is this is measured directly from the artificial medium itself and not from the individual aphids feeding on it? Figure S5(c) showed SDS PAGE gel of Sf9 crude extracts from the artificial medium, has any been done from the aphids itself?

We tried to detect P2 in aphid stylets after membrane feeding by immunofluorescence but did not succeed. In previous work, GFP-P2 was detected in aphid stylets after incubating dissected stylets with P2, while virus particles could be detected by electron microscopy in stylets of aphids having fed on infected plants or by immunofluorescence on dissected stylets (Uzest et al. 2007, doi:10.1073pnas.0706608104). Interestingly, in the same work the authors did also incubate dissected stylets with GFP-labeled P2Rev5 but did not detect a signal. We take this as further evidence that stylet binding is abolished by the mutation. What we did do to confirm stylet binding of transmissible complexes, was to place aphids after membrane acquisition of P2, P3 and virus particles on healthy turnip plants for inoculation. We observed some CaMV transmission showing that transmissible complexes had indeed bound to the stylets.

L. 391 and L. 403: This paragraph gives out two seemingly contradictory statements which reduces clarity. In L. 391, the authors states "Our membrane feeding assay support the direct effect of P2", whilst L. 403 reads as follows "Therefore, although the membrane feeding experiments do show a direct effect of P2 on aphids, interpretation of the results from the membrane feeding assays are difficult". I prefer the more cautious way the authors framed their results here in L. 403, therefore can L. 391 please be re-phrased or rewritten?

We changed the paragraph to weaken the statement: 'P2 might directly or indirectly change aphid probing behavior. Our membrane feeding assays present evidence for a direct effect of P2. Aphids having acquired different combinations of purified P2 or P3:virions through feeding on the artificial medium (thus excluding any interference from other plant or virus factors), showed altered probing behavior, compared to aphids having had access to artificial medium alone (Figure 3). Compared to inoculation feeding on healthy test plants after CaMV acquisition from infected plants (Figure 2), inoculation feeding after membrane feeding changed more behavioral parameters. We propose that this is due to the unphysiological conditions of the artificial feeding medium (high salt content, presence of detergent), necessary to maintain P2 active (Hébrard et al., 2001). Therefore, although the membrane feeding experiments do show a direct effect of P2 on aphids, interpretation of the altered behaviors is difficult.'

Reviewer #3: The correlation analysis (presented in Figure 2c) provides a tantalising hint that P2 not only modifies aphid behaviour on virus-infected plants, but may also be involved in manipulating behaviour on healthy plants that are subsequently encountered and probed. Have the authors also considered investigating a correlation between number of punctures on healthy plant (the same data plotted here) against number of intracellular punctures on the virus-infected acquisition plant? Since P2 is acquired during intracellular punctures (epidermal / mesophyll cells), this approach may reveal a more biologically meaningful effect of P2 through the comparison between the three treatment groups. I therefore recommend that this additional analysis is carried out and included in the manuscript.

We tested the correlation between the number of punctures on healthy plants against the number of intracellular punctures on acquisition plants (Mock, JI or JIΔP2) as illustrated in the following figure. However, this information does not complete our study since there is no correlation between the two parameters (cor.test, Df = 2, χ^2 = 0.607 p = 0.546). We therefore decided not to include it in the manuscript.

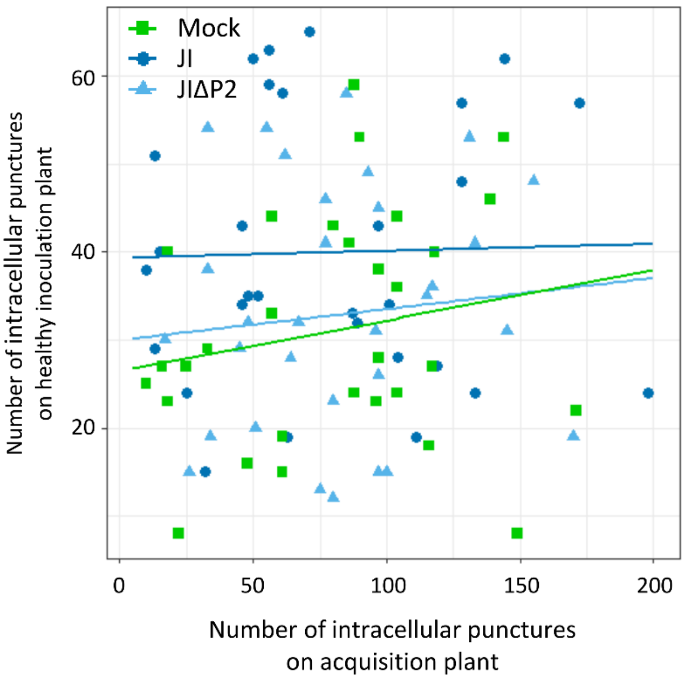

Part III – Minor Issues: Editorial and Data Presentation Modifications
Please use this section for editorial suggestions as well as relatively minor modifications of existing data that would enhance clarity.

Reviewer #1: There is clarification required in the statistical analyses. For example, the authors used Tukey for pairwise comparisons. However, I did not find any p values for this in the supplementary files, especially where differences were significant.

> *In response to your comment, we have added in the supplementary material a new table (Table S6) regrouping all the post-hoc tests (pairwise comparisons) performed when the GLM was significant. Please refer to it for the following comments on the statistical tests.*

Figure 2 Panel 'b'. It seems implausible that the pairwise comparisons for 'Intracellular punctures during first penetration' are not significant for Mock vs the mutant JIDP2. What was the p-value?

> *Indeed there was an error in the panel. The statistical test shows a difference as correctly indicated in Table S2 (0-inflated, Df = 2, $\chi^2 = 39.740$, $p < 0.001$). We have corrected the figure, thank you for noticing the mistake. The text has also been adapted to take account of this statistical effect.*

Figure 3 'penetration before first ingestion' and 'intracellular punctures' What are the p values for the pairwise comparisons between the 3 and 5 treatments, respectively?

> *Please see the new Table S6 for the p values.*

Figure 4: Panel 'c' 'intracellular punctures' What are the p values for the pairwise comparisons between the five treatments?

> *Please see the added Table S6 for the p values.*

Line 328 – do you mean 'less' in place of 'smaller'?

> *Thanks for pointing to this. The corresponding sentence was deleted during rewriting the first part of the discussion.*

Reviewer #2: Please see more detailed comments below.
The title can be edited and made more concise? Current title is "The transmission helper protein of a plant virus….". I would suggest to mention the name of the helper protein and the virus on the title directly.

> *To be more concise, the title was changed to '*The cauliflower mosaic virus transmission helper protein P2 modifies directly the probing behavior of the aphid vector *Myzus persicae* to facilitate transmission*'.*

L. 35: 'first plant contact' ? First contact with the plant

> *The wording was changed.*

L. 38: ‘virus’ would be a more fitting choice of word here rather than ‘parasite’

We changed the wording.

L. 48: ‘instantly’ might be going too far here, as it was not shown here whether it is ‘instantenous’ or not. Please re-phrase

We prefer to keep ‘instantly’ *because the behavioral changes are observed from the very first puncture into an infected plant cell, this is within seconds. This indicates that no internalization of P2, no activation of signaling pathways and the like are required, in short there is no latency.*

L.49: ‘prepares’ here personified CaMV, can this sentence be re-phrased? It is not accurate to assume that ‘CaMV prepares for..’ as what this sentence seems to be implying.

We deleted the sentence.

L. 50-51: This sentence is not clear. What does the authors mean by ‘minimizes vector implication’? Were the authors trying to say that it minimises the risk of non-transmission by the vector? Please re-phrase.

You are right, the meaning of the sentence was not clear. We wanted to say that the broad vector range of CaMV and other non-circulative viruses might be explained by the fact that no internalization of the virus (and with it complicated interactions) is required for vector transmission and manipulation. The sentence was rephrased as follows: ‘The fact that no intimate virus-vector interactions are required for vector manipulation and transmission, could explain the broad vector range of CaMV and other non-circulative viruses.’

Introduction

The introduction started with introduction to plant viruses and their mode of transmission. Followed by examples of research on virus factors which mediates changes in vector fitness. Some relevant references in this subject area are missing and should be included. Certain references displayed here are not the most accurate references available to support the statement made and ideally should be edited. Please see comments below.

L. 58: ‘their’ morphology should be ‘the stylet’s morphology’. Not the morphology of the entire aphid as an insect as what the sentence reads as at the moment.

The text was adapted.

L. 70-74: Ray and Casteel 2022 is a review paper which focused more in depth about the role of effectors to mediate plant-virus-vector interaction. The statement on L. 70 whereby “the viral factors responsible for changes in vector fitness are still largely unknown” is not entirely accurate as more has been known about viral factors affecting vector fitness. Other references are more fitting to support the statement whereby particular viral factors have been identified which are responsible to changes in vector fitness, for example in Ziebell et al., 2011; Westwood et al., 2013; Shi et al., 2016; Wu et al., 2017; Tungadi et al., 2020; Arinaitwe et al., 2022.

We replaced 'still largely' *by the weaker* 'often', *deleted the Ray & Casteel reference and added the following references: Arinaitwe et al., 2022; Westwood et al., 2013; Wu et al., 2017. We also transferred the Carr et al. 2020 reference here.*

L. 76: Donelly et al. 2019 is a paper on epidemiological modelling. Their work is still very much relevant to the theme of this manuscript. However in this manuscript, it was cited as though as it was a lab experiment paper. Donelly et al 2019 generated a modelling framework on non-persistent virus transmission where it includes aphid feeding behaviour, wingless or winged aphids, and inhibition of aphid settling on plants as parameters.

We deleted the Donnelly et al. 2019 reference because it is, as you state, a theoretic work. We deleted also the Chesnais et al. 2020 reference as it does not report on direct effects. We grouped the Carr et al. 2020 with the following references above, because it is a review on virus factors manipulating non-circulative transmission: Arinaitwe et al., 2022; Westwood et al., 2013; Wu et al., 2017.

L. 87: 'or not a' ? unfinished sentence?

We changed the sentence for more clarity. It reads now: 'One report detected altered feeding behavior on healthy test plants of whiteflies viruliferous with a non-circulative crinivirus, but since the insects were raised for two generations on infected plants before the experiments, plant-mediated effects cannot be excluded (Lu et al., 2019).'

L. 102: will explore

We added 'will'.

L. 104: 'ingest actively some cytosol' does not sound right, please re-phrase this sentence.

We replaced 'cytosol' *by* 'cellular contents' *because aphids might also ingest vacuole sap and organelles.*

L. 104: 'if the plant is accepted' , please re-phrase this sentence as well.

We replaced 'accepted' *by* 'susceptible' *and added a comma after* 'tissues'.

L.106-107: ingest phloem sap passively and continuously.

We changed the wording.

L. 102-111 : This paragraph can be shortened. I understand that the authors are trying to describe the sequence of events that occurred after the aphid lands on the plant which is relevant to the paper.

The entire paragraph was revised and reads now: 'Aphids landing on a new plant will explore the plant's suitability with test punctures. For this, they insert the stylets in epidermis and mesophyll cells, salivate briefly into the cytoplasm and ingest actively some of the cellular contents. If the plant is susceptible, the stylets advance deeper into the tissue, doing more test punctures until they are inserted into the sieve tubes. Here the feeding behavior changes: after an active salivation phase, the aphids ingest phloem sap passively and continuously, their principal food source. Because P2 locates

exclusively in infected cells, it can be acquired only during test punctures, whereas P3:virions can be acquired during test punctures and phloem sap ingestion (Palacios et al., 2002).*'*

The authors then settled to choose P2 to test their hypothesis whether a non-circulative virus like CaMV can encode factors having a direct effect on the vector
L. 121-122 : Why only P2 and not P3 as well?

*P2 alone is sufficient to bind to the stylets. P3 is an adapter protein linking P2 to the virus particle, it does not interact directly with the aphid and is therefore less likely to be involved in modifying the behavior of the vector. We changed the last sentence of the introduction for more clarity: '*We chose P2 to test for this hypothesis, because it contains the interaction domain for binding to the aphid stylets, making it an excellent candidate.*'*

Results
L.124: The title of this subsection does not really make sense, particularly in the part of "P2 probing". Please re-phrase.

*You are right. The title was changed to: '*Aphid feeding behavior is different on plants infected with wild type CaMV expressing P2*'*.

Fig 1a : can the authors add labelling on the figure itself please?

Nice idea! We added labels to the leaves in Figure 1a and also in Figure 4a.

Fig 1b : can the authors check if the labelling is correct on the western blot? If anti P2 is an antibody specific for P2 protein, then why does bands appear on JIΔP2 samples?

We corrected the labeling (see also above).

The main take home message is number of intercellular punctures made by the aphids increased significantly during JI (wild-type) virus infection but not on plants infected by JIΔP2 virus.
L. 148: The authors then went on to test the effect of P2 on aphids using wild-type CaMV and P2 deletion mutant, JIΔP2. Deletion of P2 does not affect infectivity of the virus. They did EPG and found that deletion of P2 also does not affect on phloem sap ingestion by the aphids.
However the number of intracellular punctures were a lot higher on JI-infected plants than JIΔP2 or mock inoculated ones. This section was summarised nicely on L.179-182 where they stated that CaMV infection (with and without P2) did increase phloem feeding and reduce the time taken by the aphids to reach the phloem.

Thank you!

L. 178-182: They also stated that the number of intercellular punctures were higher on aphids fed on wild-type CaMV Ji virus but not on the mutant virus or mock inoculated plants. The closing statement from this section where the authors indicated that 'P2 was responsible for this' sounds premature from the conclusion of this work to date. This work here does not exclude the possibility that the observed effects can be the due to alteration in the host plant, thus an indirect effect of CaMV.

This is correct and it is the reason why we carried out the membrane feeding experiments.

Have the authors tested whether aphids were able to acquire the mutant CaMV JI-P2Rev5 virus? The authors did demonstrate that turnip plants were infected with CaMV JI-P2Rev5 virus, but with P2 being unable to bind to the aphid stylet – have the authors confirmed that aphids can acquire CaMV JI-P2Rev5 ?

Please see our prior response in Part II Major issues.

L. 183: The title for this subsection is not entirely accurate. Please re-phrase and be more specific, i.e: mention P2 protein on the title?

We tried to improve the title. It reads now: 'Post-acquisition effect of P2-expressing wild type CaMV on aphid feeding behavior'

L. 227 – 228: The title for this subsection on "Direct interaction between P2 and Myzus persicae modifies the probing behaviour of the aphid vector" needs to be re-phrased as the authors have not shown protein-protein interaction between P2 and aphid stylet here in their results. It would be more accurate to re-name the subsection as "Acquisition of P2 virions (and/or P3 virions) xxx by M. persicae modifies xxx ".

We changed the section title to 'Feeding on purified P2 and virus particles modifies aphid probing behavior'*.*

L. 265-266: Similar to my previous comment. The title for this subsection on 'The interaction between P2 and the aphid stylets is required for changing probing behaviour' needs to be re-phrased as the authors have not shown a direct protein-protein interaction here between P2 and the aphid stylets. It would be more accurate to re-name the subsection as "Acquisition or feeding from xxx ".. or "Aphids feeding on mutant virus with abolished function of P2 interaction to xxxx ".

The title was rephrased to 'Feeding on plants infected with a CaMV mutant expressing a P2 defective in stylet binding abolishes changed aphid probing behavior'*.*

L. 287: 'interacting with intestine'? please re-phrase. Did the authors meant to say interacting with host factors in the aphid gut/intestine?

*In response to your comment and to a suggestion by reviewer 3 who pointed out that P2 could bind also to yet other parts of the digestive system, we changed the sentence to'…*interacting with vector factors in the more posterior parts of the digestive tract.*'*

Figure 2 : has the authors showed here that aphids can acquire JIΔ2P?

Like for CaMV JI-P2Rev5 (see our response in Part II), we did not test formally, using membrane feeding assays, whether aphids can acquire and transmit CaMV JIΔP2 virus particles when wild type P2 is added to the feeding medium. However, since the mutant codes for wild type coat protein and P3, CaMV JIΔP2 particles should be transmitted under this condition, just as are other non-transmissible CaMV mutants.

L. 301: performed more than twice

We changed the wording.

L. 315: the authors did acknowledge that 'the acrostyle-binding capacity for P2 is important for this'. Hence I think it is important that the authors demonstrate whether aphids can acquire and subsequently able to transmit CaMV JI-P2Rev5 from a CaMV JI-P2Rev5 infected plants or not. This is important as otherwise, the observed aphid behaviour when feeding on CaMV JI-P2Rev5 infected plants will be due to an indirect host-mediated effect whether the aphids were responding to virus-mediated effect to the plant hosts.

*Our previous work shows that the CaMV CabbS isolate harboring the P2Rev5 mutation was not transmissible in plant-to-plant transmission experiments (Moreno et al. 2005 doi:10.1128/jvi.79.21.13587-13593.2005). In addition, in vitro binding experiments using dissected stylets showed that recombinant P2Rev5 did not bind to stylets (Uzest et al. 2007, doi:10.1073pnas.0706608104). Taken together, this is evidence that it is stylet binding of wild type P2 that induces the observed behavior changes. A sentence was added to indicate this: '*This is supported by the fact that a CaMV isolate harboring the P2Rev5 mutation is not transmissible (Moreno et al., 2005) and that recombinant GFP-tagged P2Rev5 does not bind to dissected stylets in in vitro interaction assays (Uzest et al., 2007).'

L. 340-341: Is there any reference that can be cited which shows that phloem contained higher virus titer than the surrounding single cells?

To our knowledge, there is no data published on the CaMV titer in the phloem sap. From electron microscopy images of plants infected with CaMV (Palacios et al, 2002 doi: 10.1099/0022-1317-83-12-3163) or with the related dahlia mosaic virus (Kitajima et al. 1969 doi: 10.1016/0042-6822(69)90044-0), it seems that there is less virus in the phloem than in surrounding cells. What we wanted to indicate is that the virus diversity might be higher in the phloem (input from many cells) than in cells (should contain a more or less clonal virus population). However, because this is purely speculative, we chose to delete the corresponding second half of the sentence: '~~especially because the phloem has input from the surrounding tissues and might contain a more diverse virus population than do single cells.~~'

L. 338-345: This paragraph is confusing, does the authors meant to say that P2 is not involved in the alterations of aphid behaviour (i.e: lower number of cell punctures) as they observed a similar effect on aphids fed on both wild-type CaMV inoculated plants and CaMV P2 deletion mutant inoculates plants? This somewhat contradicts the results from Fig.1 where on L. 172-175, it states that 'we observed P2-speficic alterations for the number of intracellular punctures during the first probe which was twice as high on JI-infected plants than on JI P2-deletion mutant infected plants'. This is also re-iterated again on L. 347-348. Please edit this paragraph.

Three behavior features were changed. First, longer phloem acquisition on infected plants (JI and JIΔP2). Second, aphids did less intracellular punctures in infected plants than on healthy plants during the entire observation period (8 hours), irrespective of the presence of P2. However, third, the number of test punctures during the first penetration was doubled in wild type-infected plants, i.e. in the presence of P2. We try to state this more clearly in a thoroughly revised first part of the discussion on behavior changes and their impact on transmission. We also shortened this part considerably to concentrate on the main points: 'Analysis of feeding behavior on infected plants showed significant modification of three feeding activities that are related directly with

CaMV acquisition (Figure 1). One, the duration of phloem ingestion increased, two, the total number of test punctures during the entire eight hour observation period decreased, while, three, their number during the first stylet penetration doubled.

Prolonged phloem ingestion might be advantageous for CaMV transmission because the phloem sap contains virus particles and uptake of CaMV from the phloem has been reported before (Palacios et al., 2002). Prolonged phloem ingestion was observed previously with the same CaMV isolate on two other hosts, *Arabidopsis thaliana* and *Camelina sativa* (Chesnais et al., 2021). This could therefore highlight potential adaptive virus effects. The second observation, reduction of intracellular test punctures over the entire observation period, might be considered counterproductive to acquisition because it should decrease chances to acquire P2, which is absent from the sieve tubes. However, the work by Palacios and coworkers showed that combined uptake of CaMV from tissue and phloem sap might even be more efficient than uptake from epidermis and mesophyll cells alone. Thus, potential negative effects of decreased intracellular test punctures seem to be outweighed by earlier and longer phloem ingestion. This differentiates CaMV from typical non-circulative viruses that are acquired during test punctures in the tissue and lost rapidly when vectors reach the phloem (see for example (Wang and Ghabrial, 2002). These two parameters, phloem ingestion and total number of test punctures, were altered similarly for aphids feeding on plants infected with JI and with JIΔP2. This demonstrates that P2 plays no role in this and that other viral factors are involved. A candidate is P6-TAV, which contributes to the modification of aphid feeding behavior although it alone is not sufficient to induce all behavior changes (Chesnais et al., 2021).

The third parameter, the number of test punctures during the first stylet penetration, was doubled on plants infected by JI, but not on healthy plants or those infected by JIΔP2. As removing the coding sequence of P2 from the CaMV genome did not affect other parameters of the infection (Figure 1), we suggest that the increase in test punctures during the first penetration may be caused exclusively by P2. Test punctures are mandatory for acquisition of P2 (which is contained only in epidermis and mesophyll cells (Palacios et al., 2002)) and their increase during the first stylet penetration might speed up P2 acquisition and consequently the acquisition of P3:virions. Virus acquisition during the first stylet penetration is also favorable for virus transmission by non-host aphids that reject incompatible plants after the first test punctures and leave them (Fereres et al., 1993; Pitt et al., 2022). Carmo-Sousa et al. (Carmo-Sousa et al., 2014) report similar results as here for the non-circulative cucumber mosaic virus (CMV). They observed that aphids exhibited during the first 15 minutes – but not later – twice as many test punctures on CMV-infected plants than on mock-inoculated plants. Therefore, this change in aphid behavior during its first contact with the infected plant may be very important for the acquisition of viruses transmitted in a non-circulative manner like CaMV and CMV. An interesting point is that the behavior change is in both cases immediate since it is observed from the very first test punctures onwards. This suggests that the 'active compound' does not need to traverse the intestine to induce behavior modification.'

L. 347: Is there more than one CaMV isolate used in this study?

We only used JI and two mutants derived from it, JIΔP2 and JI-P2Rev5, in this study.

L 357 : Has there been studies to show whether the level or titer of P2 protein on the stylet remained the same throughout ? whether aphids for example had higher titer of P2 protein on their stylet tips during the initial acquisition, which then increased and tapered off?

There is no information available on the level of P2 in stylets over time. What is known, is that P2 remains functional in the aphids over a period of at least several hours.

L.364-367 contradicts L. 338-339, please check and edit.

This comment is similar to your comments above. As indicated there, there are two observations. One is that over the entire 8 h period of observation the number of test punctures diminished, the other is that during the first stylet penetration twice as much test punctures were made. We hope to have made this difference clearer in the new version of the discussion.

L. 389: The opening sentence where "We show here that P2 changes aphid probing behaviour.." again does not sit comfortably with me as the authors have not shown yet whether the effect was direct or indirect. Rather than addressing the 'direct or indirect' effect in the second sentence, it would be better if the authors opens this paragraph by stating the fact directly "We show here that P2 may directly or indirectly changes……."

*In response to your comment in Part II, the corresponding paragraph was rewritten and we hope it is now easier to understand. Concerning your suggestion to add 'directly or indirectly' to the opening phrase, it reads now: '*P2 might directly or indirectly change aphid probing behavior.*'*

L. 391 and L. 403: This paragraph gives out two seemingly contradictory statement which reduces clarity. In L. 391, the authors states "Our membrane feeding assay support the direct effect of P2", whilst L. 403 reads as follows "Therefore, although the membrane feeding experiments do show a direct effect of P2 on aphids, interpretation of the results from the membrane feeding assays are difficult". I prefer the more cautious way the authors framed their results here in L. 403, therefore can L. 391 please be re-phrased.

We responded to this concern in Part II. Please see there.

Did the authors measure the success rate of subsequent virus inoculation onto healthy plants by the aphids after being fed on the membrane? Did the authors also detect and measure P2 and/or P3 protein presence on the aphids after they were fed on the membrane to verify that the effects observed were genuinely due to the P2 and/or P3 proteins being taken up by the aphids and not just due to aphids experiencing feeding difficulties on the membrane.

We addressed these issues in Part II. Please see there.

L. 175 – 176 and L. 339-342 : Both set of sentences also seems to convey contradictory message whereby on L. 175-176, the authors stated that the number of intracellular punctures during the first probe was twice as high on JI-infected plants than on JIdP2 infected ones or mock-inoculated ones. This seemingly contradict the statement on L. 339-344 where the authors stated that P2 plays no role in this as the observed the same behaviour on plants infected with wild-type virus and on plants infected with JIdP2.. whereby "this" was mentioned in L. 339 as "lower number of test punctures combined with the longer phloem ingestion on infected plants". Implying that on L. 175 : aphids made more punctures on JI-infected than

on JIdP2 infected plants, however on L. 339-342 : aphids showed similar number of test punctures on both JI- and JIdP2 infected plants.
This is either a grammatical error or the sentences need to be restructured as to avoid confusion to readers.

We hope that the revised discussion dealing with aphid behavior differences is easier to understand and to read. Please see also our responses above.

Reviewer #3: I have a general issue with the term 'feeding behavior' (e.g. in the heading line 124 and numerous other places in the text) to indicate all behaviours displayed by aphids during stylet penetration. On plants, only E2 (sustained phloem sap ingestion) is really "feeding". Better to use more generic ('stylet penetration' / 'probing') alternatives throughout the manuscript.

We try to use throughout the text the term 'feeding' for phloem sap ingestion and 'probing' or 'testing' for intracellular punctures.

The authors seem to over-state the evidence for causative links in some parts of the text. I have suggested more cautious language in parts, and made some other specific comments with line numbers indicated as follows.

Line Comment

50 The meaning of 'minimises vector implication' is not clear, what is the significance?

*Indeed, the meaning of the sentence is not clear and also Reviewer 2 noted this. The sentence reads now: '*The fact that no intimate virus-vector interactions are required for vector manipulation and transmission, could explain the broad vector range of CaMV and other non-circulative viruses.*'*

56 'Hemiptera' is an order (so not italics).

We changed the style.

94 'fixation' implies a non-reversible link - change to 'adherence'.

Thanks for noting this. Text was adapted accordingly.

108, 110 'tissue cells' – can a more useful and specific term be used? Peripheral? Phloem sieve elements are part of a (phloem) tissue, so 'tissue and from phloem sap' does not make sense

*The sentence in question was reformulated for more clarity: '*Because P2 locates exclusively in infected cells, it can be acquired only during intracellular test punctures, whereas P3:virions can be acquired during test punctures and during phloem sap ingestion (Palacios et al., 2002).*'*

125, 158 The statement does not agree with Figure 1 b), which indicates that P2 is not detected in WT plants – has the figure been mislabelled?

Thanks for noting this. We corrected the labeling. We also introduced label for the leaves in Figures 1a and 4a, as suggested by Reviewer 2.

125 Figure 1 c) & d) and subsequent figures need full axis labels – presumably duration (hours) and occurrence (number of events per recording)?

We corrected the axis labels to display parameter and unit.

Note spelling of 'occurrence' (two r's). What is being plotted – means and standard errors? This also needs clarifying in subsequent figures.

The spelling of 'occurrence' was corrected. The missing information (means and standard error) was added to the figure legends.

160 Change 'taking' to 'taken'

We corrected the wording.

167 Change '1a' to '1c'

Thanks for noting the mistake, which we corrected.

178 Change to '….without P2) was associated with an increase in the duration….' (there's no basis for a causal link at this point).

*We replaced '*with and without P2*' by '*JI and JIΔP2*'.*

179 'reduce the time required to reach it' is not supported by the data and should be deleted, unless it can be evidenced. Figure 1c) shows no significant difference (time to first sap ingestion).

You are right. The data do not show significant differences. We deleted the part of the sentence stating this.

182 Change to '… was associated with this.' (again, no basis for a causal link at this stage).

The sentence was modified accordingly.

187 Change 'lists' to 'presents'.

We changed the word.

215 Change 'was' to 'may be'.

The word was changed.

287 I suggest changing 'intestine' to 'more posterior parts of the alimentary canal' (or something similar). There seem to be lots of other possibilities for potential interaction sites (other than the intestine).

You are right. The part of the sentence reads now: 'interacting with vector factors in the more posterior parts of the digestive tract'*.*

306 Change 'only on the rim of significance' to 'marginally not significant'.

The wording was changed to 'marginally insignificant'*.*

315 Change 'this' to 'the observed changes in vector behavior' (or similarly expand this rather brief last sentence).

The beginning of the sentence was changed to: 'The observed behavioral changes indicate…'

316 Delete 'a'

The word was deleted.

326 Change 'behavior' to 'behavioral'.

The word was changed.

328-330 Can these statements ('aphids localize the phloem faster'/'faster access to the phloem') be supported by reference to specific parameters and statistical outcomes? Time to first E1 / E2?

This part of the discussion was thoroughly restructured and rewritten and these statements are not part of it anymore.

352 Change 'is' to 'may be'.

The word was changed in the rewritten discussion.

362 Change 'did' to 'exhibited'.

The word was changed in the rewritten discussion.

393 Change to 'altered subsequent probing…'

The word was added in the rewritten discussion.

433 Suggest changing to 'is not definitive evidence'. Otherwise this sentence does not make sense, particularly when the next sentence allows for other hypotheses.

We believe that 'evidence' indicates a possibility and not a fact. We modified the following phrase and added 'also' to it to indicate that both possibilities may apply: 'P2 might also exercise an effect by binding further down in the digestive tract.'

434 Again, it seems strange to highlight particular regions of the alimentary canal (cibarium, intestine) when many other potential binding sites are possible.

We changed the wording to ‘digestive tract’.

454 Change ‘inversed’ to ‘distinctly different’.

The word was changed.

502 Change ‘depression’ to ‘negative air pressure’.

The word was changed.

509 Change aphids to aphids’.

The wording was adapted.

556, 566, 586 Change ‘in’ or ‘by’ to ‘previously’.

The wording was changed.
