# Supplementary material for: The cauliflower mosaic virus transmission helper protein P2 modifies directly the probing behavior of the aphid vector Myzus persicae to facilitate transmission
Source: PLoS Pathog. 2023 Feb 6;19(2):e1011161. doi: 10.1371/journal.ppat.1011161 (PMC9934384; doi:10.1371/journal.ppat.1011161)
Supplement: Attachment — Submitted filename: Reviewer Comments .pdf [file ppat.1011161.s015.pdf]

There is clarification required in the statistical analyses. For example, the authors used Tukey for pairwise comparisons. However, I did not find any p values for this in the supplementary files, especially where differences were significant.

Figure 2 Panel ‘b’. It seems implausible that the pairwise comparisons for ‘Intracellular punctures during first penetration’ are not significant for Mock vs the mutant JIDP2. What was the p-value?

Figure 3 ‘penetration before first ingestion’ and ‘intracellular punctures’ What are the p values for the pairwise comparisons between the 3 and 5 treatments, respectively?

Figure 4: Panel ‘c’ ‘intracellular punctures’ What are the p values for the pairwise comparisons between the five treatments?

Line 328 – do you mean ‘less’ in place of ‘smaller’?
